# Effects of the DAGIS randomized controlled trial on home environment and children’s food consumption according to the degree of implementation

**DOI:** 10.1186/s12889-022-14639-y

**Published:** 2022-12-05

**Authors:** Reetta Lehto, Henna Vepsäläinen, Aku-Ville Lehtimäki, Elviira Lehto, Marja H. Leppänen, Essi Skaffari, Anna M. Abdollahi, Eva Roos, Maijaliisa Erkkola, Carola Ray

**Affiliations:** 1 Folkhälsan Research Center, Topeliuksenkatu 20, 00250 Helsinki, Finland; 2 Department of Food and Nutrition, University of Helsinki, P.O. Box 66, 00014 Helsinki, Finland; 3 Faculty of Social Sciences, Department of Sociology, University of Helsinki, P.O. Box, 00014 Helsinki, Finland; 4 Faculty of Educational Sciences, Department of Teacher Education, University of Helsinki, P.O. Box 9, 00014 Helsinki, Finland; 5 Faculty of Medicine, University of Helsinki, Helsinki, Finland; 6 Department of food studies, Nutrition and Dietetics, Uppsala University, Uppsala, Sweden

**Keywords:** Process evaluation, Preschooler, Food intake, Sugar intake, Fruit and vegetables, Sugary drinks

## Abstract

**Background:**

Combining process evaluation data with effectiveness data and examining the possible mediators of intervention effects elicits valuable knowledge about how and for whom these interventions are effective. The aim of this study was to examine whether the parental degree of implementation (DOI) of a home-involving preschool intervention affected children’s food consumption via home mediators.

**Methods:**

The five-month Increased Health and Wellbeing in Preschools (DAGIS) intervention involved 476 participating children aged 3–6 years and was conducted in 2017–2018. Parents reported children’s food consumption (g/day) outside childcare hours, the availability of foods at home, role modelling of food consumption, and the norms related to food consumption. In addition, parents reported the extent to which they had implemented the intervention program at home. Mediation analyses were conducted to examine the effect of low and high DOI compared to control group on the change in children’s consumption of fruit and vegetables (FV), sugary everyday foods, sugary treats, and sugar-sweetened beverages (SSB) via food availability in the home, parental role modelling and parental norms.

**Results:**

Compared to the control group, there was a direct effect of a high DOI on diminishing consumption of SSB (B -27.71, 95% CI -49.05, -4.80). No indirect effects were detected. In the high DOI group, a change in parental norm was associated with increased FV consumption showing an indirect effect (B 4.31, 95% CI 0.23, 10.59). In the low DOI group, there was an indirect effect via decreased food availability leading to decreased sugary everyday food consumption (B -2.17, 95% CI -5.09,  -0.09).

**Conclusions:**

Combining process evaluation and effectiveness data revealed a decrease in children’s SSB consumption only in the high DOI group, as well as indirect effects on children’s consumption of FV and sugary everyday foods. In order to gain more intervention effects, further studies are required in order to examine parental facilitators and barriers to the implementation of interventions and how to impact effectively the determinants of the targeted behavior.

**Trial registration:**

ISRCTN57165350 (8 January, 2015).

**Supplementary Information:**

The online version contains supplementary material available at 10.1186/s12889-022-14639-y.

## Background

Like adults, children in many Western countries consume less than the recommended amount of fruit and vegetables (FV) [1–3]. An abundant consumption of FV is recommended in order to gain health benefits, such as the prevention of cardiovascular diseases, diabetes and cancer [4–6], and to increase sustainability of food consumption [7]. Furthermore, many studies have shown that the intake of added sugar is high among children [8, 9]. A high intake of added sugar is associated with lower overall quality of diet [8, 10] and the consumption of sugar-sweetened beverages (SSB) may be a predictor for higher weight gain among children [11]. Similarly to other Western countries, the prevalence of overweight among Finnish children and adults is high (27–28% and 50–62% respectively) [12, 13]. In addition, dietary habits formed in early childhood often track later into a child’s life, even into adulthood [14, 15]. Thus, promotion of abundant FV consumption and limited added sugar intake among young children is well founded and may have long lasting benefits for children’s diet and health.

Studies on the promotion of healthy food consumption among young children show mixed results. A meta-analysis of home and/or early childhood education and care (ECEC) setting interventions, aiming to increase FV consumption among children aged 5 and under, found little evidence of intervention effectiveness [16]. Home, ECEC and school setting interventions aimed at decreasing SSB consumption among children, on the other hand, have shown some positive effects [17, 18]. In addition, a systematic review examining intervention effects on children’s energy balance-related behaviors (EBRB) in ECEC settings found that most interventions had a small positive effect on children’s food consumption, which frequently included an increase in FV and a decrease in SSB consumption [19].

When promoting a healthy diet among young children, the determinants of food consumption are of crucial importance, as children themselves have only a limited impact on what they eat. Both social and physical home food environment, such as parenting practices and food availability and accessibility, are important determinants of food consumption among preschool-aged children [20]. The availability of both healthy and unhealthy foods at home, as well as role modelling of eating, are associated with a higher consumption of these foods among children [21, 22]. In addition, impacting food availability has been found to be an effective intervention method in changing children’s food consumption [23]. There has been less research into parental norms regarding children’s food consumption, meaning what parents think is a normative amount or way for children to consume specific foods or drinks, but at least one study has examined adolescents’ subjective norms (what they consider others want them to consume) in relation to their soft drink consumption [24]. Parental norm could act as an important predictor of parents’ restrictions or encouragement of the consumption of specific foods or drinks among children.

In order to gain more knowledge about how and for whom health behavior interventions are effective, the role of determinants and intervention implementation should be considered and systematic ways to develop and evaluate interventions should be used. Intervention Mapping is a standardized method of developing health promotion interventions, and it stresses the importance of knowing and impacting the determinants of the targeted health behavior [25]. In addition, process evaluation data elicits information about whether an intervention was implemented as intended. The effectiveness of an intervention can depend on the extent to which it was actually implemented and this can vary greatly between participants [26]. Some [18, 26–29], but not all [30] studies on health behaviour interventions show that higher implementation rate associates with intervention effects whereas intervention groups with low degree of implementation (DOI) might not differ from control group.

The DAGIS study, which aimed to promote healthy EBRBs and self-regulation skills among children in ECEC settings, used Intervention Mapping in the program development [31]. In that process, we defined our main determinants of children’s EBRBs in the logic model of change based on previous results and a literature review [29]. Concerning food consumption, these main determinants were food availability at home, parental role modelling of eating, and parental norms regarding children’s food consumption among others. A need for information about change mechanisms in interventions aiming to promote children’s health behaviors has been acknowledged [32, 33].

Earlier, we have reported that the DAGIS intervention had no effect on children’s consumption of FV or sugary foods and drinks when measured in terms of consumption frequency [34]. However, some effects were found on the home environment related to food consumption [35]. Given the need for knowledge about change mechanisms of health behavior interventions and the important role of DOI found in previous studies, we wanted to examine the role of the DOI and mediated intervention effects in the DAGIS intervention.

The aim of this study was to examine whether the parental DOI of the DAGIS intervention had an effect on 3–6-year-old children’s food consumption (namely consumption of FV, sugary everyday foods, sugary treats and SSB) and was the effect mediated by a change in the possible home environment mediators of food consumption (the availability of foods, parental role modelling, norms).

## Methods

The intervention was part of the Increased Health and Wellbeing in Preschools (DAGIS) study, which examines children’s EBRBs, their determinants at home and at ECEC centers and the socioeconomic differences between them [36]. The DAGIS intervention promoted healthy EBRBs and self-regulation skills among children in ECEC centers via a 5-month family-involved ECEC setting intervention. The prospective trial registration number of the intervention is ISRCTN57165350 (8 January, 2015). The DAGIS intervention study was reviewed by the Research Ethics Committee in the Humanities and Social and Behavioural Sciences of University of Helsinki (22/2017; 16 May 2017) and was found ethically acceptable. The study was performed in accordance with the Declaration of Helsinki. Participant recruitment has been thoroughly described in an earlier article [34]. In short, two municipalities in Southern Finland participated in the intervention. In one municipality, all municipal (public) ECEC centers (*n* = 28) participated in the study. In the other municipality, 3 volunteering ECEC centers participated. Families with children aged 3–6 years were recruited for the study via the ECEC centers. The parents of 802 children gave written informed consent, the participation rate being 47%.

**Fig. 1:**
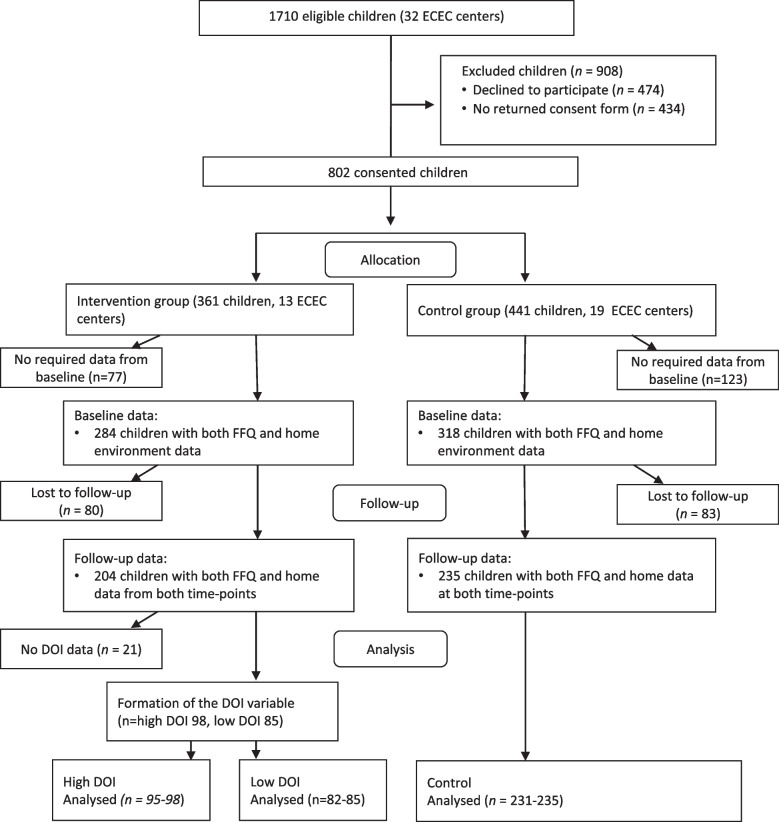
Flow chart of DAGIS intervention participants and the analytic sample ECEC = early childhood education and care; FFQ = food frequency questionnaire; DOI = degree of implementation

Baseline measurements were conducted in September–October 2017, prior to the randomization of the ECEC centers into the intervention or the control group. The intervention was parallel with 1:1.2 allocation ratio*.* This allocation ratio was decided as we expected the drop-out rate to be higher in the control group, who did not have any contact with the research team between the measurements. Follow-up measurements were conducted in April–May 2018 and included all the same measurements from the baseline. In addition, process evaluation of the intervention was assessed at follow-up by parental self-report. The intervention lasted from mid-November to the beginning of April. The analytic sample of this study consisted the 439 children (55% of all study participants), who had valid data on the outcome and mediator variables from both baseline and follow-up. The flow chart of participants and the analytic sample is shown in Fig. 1.

### Intervention content

The intervention was carried out at ECEC centers by the ECEC personnel and at home by parents. It was based on a program promoting children’s self-regulation skills [37], and health behavior themes such as food consumption, physical activity, and screen time were added to it. The self-regulation activities were mainly implemented in ECEC centers, whereas the health behavior activities developed for this study were mainly implemented at home by parents. The intervention has been more thoroughly described elsewhere [31]. It was divided into five periods, all of which had distinct themes: 1) self-regulation skills, 2) physical activity, 3) FV, 4) screen time, and 5) sugary foods and drinks. Each period lasted 4–6 weeks and included components to be implemented at the ECEC center and at home. In this study, we use process evaluation data mainly from periods 3 and 5, which had a food theme. The program content for parents during these periods comprised: an information letter containing tips for parents on how to impact child’s FV and sugary food and drink consumption; a story encouraging children to try new flavors, to be read and discussed with the child; a family activity game with food-related activities, and a personal feedback letter from the research team on their child’s FV and sugary food consumption based on baseline measurements. Parents also received e-mails on the topic of the given period sent by the research team via ECEC centers. In addition, the ECEC centers were meant to organize one parental afternoon/morning during each period that included activities for child/parent-dyads on the period’s theme.

### Exposure: degree of implementation (DOI)

We collected process evaluation data from ECEC center managers, personnel and parents, according to Saunders’ process evaluation framework and the RE-AIM model [38, 39]. Four aspects of program implementation were assessed: dose delivered; dose received – exposure; dose delivered – satisfaction, and fidelity. Reach was not included in the DOI variable. We considered filling in the follow-up questionnaire to represent reach: those from whom we had process evaluation data had been reached and thus those not reached are not included in the analyses. As the topic of this study was the home environment, we only used parental process evaluation data in this study.

Parental process evaluation data was collected via a questionnaire during the follow-up measurements. We formed a variable on the DOI as a sum of questions assessing dose delivered, dose received – exposure, dose delivered – satisfaction, and fidelity. Many of the questions were based on the RE-AIM model and adapted from the ToyBox Study [39, 40]. The questions and their scoring are shown in Table 1. The questions included concerned either the whole intervention or specifically periods 3 and 5, which had a food theme. We stressed the dose received – the exposure dimension in the scoring – as we wanted to put weight on parents’ active implementation of the intervention. The scale of the sum variable was 0.45–18.5 points, the mean being 10.0 points (SD 3.3). The sum variable was then dichotomized from the median (10.1 points) in order to create two groups: low DOI and high DOI. In the analyses, these groups were compared against the control group.

**Table 1:** Questions included in the degree of implementation (DOI) variable and their scoring

Questions	Answer options	Scoring
**Dose delivered,** 0–3.5 points		
1. Are you aware of the following DAGIS materials or contents? a. DAGIS letter and content related to vegetables and fruit b. DAGIS letter and content related to sugar c. Tiger Star Eye character made by the child at preschool d. DAGIS greetings to families -emails e. Feedback on the child’s health behaviors f. DAGIS bulletin board at preschool g. DAGIS intra website	Yes/no	No: 0 points Yes: 0.5 points
**Dose received – exposure,** 0–9 points		
1. Have your family implemented the following bingo tasks? a. Bingo related to fruit and vegetables b. Bingo related to sugary foods and drinks	1 = I am not aware of the bingo 2 = I have not been familiarized with the bingo 3 = I have not implemented tasks, but we have familiarized with the bingo 4 = Yes, we did the tasks, but we did not get a bingo 5 = Yes, and we got a bingo	1–3: 0 points 4–5: 1 point
2. Have your family read the following DAGIS materials? a. Story on the courage to taste new foods b. The materials in the DAGIS letter on fruit and vegetables c. The materials in the DAGIS letter on sugar d. DAGIS greetings to families -emails e. Feedback about the child’s health behaviors	1 = I am not aware of the material 2 = We haven’t read it at all 3 = Yes, we have read it a bit 4 = Yes, we have read it once 5 = Yes, we have read it several times	1–3: 0 points 4–5: 1 point (except b. and c.: 0.5 points for answer options 4–5)
3. Did you or the other possible guardian participate in parental afternoons/mornings in your child’s preschool? a. The event in January b. The event in March/April	1 = Yes 2 = No 3 = I was not aware of the event	1: 2 points 2–3: 0 points
4. Have you discussed with your child how you can help the DAGIS characters to make their health behaviors healthier?	Yes/No	No: 0 points Yes: 1 point
**Dose received – satisfaction,** 0.15–3 points		
1. In my opinion, the written DAGIS materials were … a. easy to read b. had too much information for me (reverse coded)	1 = Strongly disagree 2 = Somewhat disagree 3 = Neither disagree or agree 4 = Somewhat agree 5 = Strongly agree	1–3: 0 points 4–5: 1 point
2. The DAGIS project was … a. useful for our family b. motivated our family to promote the child’s health behaviors c. had too many activities to implement (reverse coded)		
3. Overall, what did you think about the DAGIS project?	1 I did not like it at all – 5 I liked it a lot	0.3 points per self-rated grade
**Fidelity,** 0.3–3 points		
1. In your opinion, how well did your family implement the tasks, activities and tips of the DAGIS project?	Give a grade on a scale 1–10	0.3 points per self-rated grade
**In total 0.45–18.5 points**		

### Mediators: food availability, parental role modelling and parental norms

Parents filled in a questionnaire on the child’s home environment related to food consumption. Test-retest reproducibility of the home environment questions related to children’s food consumption have been tested, showing moderate to good reproducibility [41]. Three possible mediators of the intervention effect on children’s food consumption were used in this study: 1) food availability at home, 2) parental role modelling, and 3) parental norms relating to children’s food consumption. Each of these mediators were specific for the four food consumption variables used as outcomes, e.g. availability of SSB was used as a mediator when the outcome was the child’s SSB consumption. The questions and formation of the variables used in the analyses are shown in Table 2. Differences in follow-up and baseline values were used in the analyses to represent changes in the mediators.

**Table 2:** The formation of the mediator variables

Home environment	Questions	Food items	Answer options	Formation of variables	Variable used in analyses
Availability of foods at home	How often have you had the following foods at home during the last month?	FV: fresh vegetables; fresh fruit or berries; frozen vegetables; and frozen fruit or berries (asked separately)	1 = Never; 2 = Rarely; 3 = Sometimes; 4 = Often; 5 = Always	Mean of 4 items	Difference in follow-up and baseline value
		Sugary everyday foods: cereals or muesli with added sugar; yogurts with added sugar; puddings, quarks etc. with added sugar; berry soups (asked separately)		Mean of 5 items	
		Sugary treats: sweets or chocolate; cookies; sweet pastries; ice cream (asked separately)		Mean of 3 items	
		SSB: soft drinks with added sugar; juices with added sugar (asked separately)		Mean of 2 items	
Parental role modelling of food consumption	During the past week, how often did you consume the following foods, when your child was around?	Fruit and vegetables (asked separately)	Not once; 1–2 times/week; 3–4 times/week; 5–6 times/week; daily; More than once a day	Conversion into 0, 1.5, 3.5, 5.5, 7, and 10.5. Mean of two items	Difference in follow-up and baseline value
		Sugary everyday foods^a^ (asked as one item)			
		Sugary treats^b^ (asked as one item)		Conversion into 0, 1.5, 3.5, 5.5, 7, and 10.5, used as a continuous variable	
		SSB^c^ (asked as one item)			
Parental norms	I think the suitable amount of X foods/drinks for 3–6-year-old children is X portions a day or X portions a week	Suitable number of portions of vegetables/fruit (asked separately)	Open question	Fruit and vegetables summed up, conversion to portions per day	Difference in follow-up and baseline value
		Suitable number of portions of sugary everyday foods^a^			
		Suitable number of portions of sugary treats^b^ Suitable number of portions of SSB^c^		Conversion to portions per day	

*FV* Fruit and vegetables, *SSB* Sugar-sweetened beverages

^a^Examples given: yogurts, puddings, breakfast cereals, and muesli with added sugar

^b^Examples given: candy, chocolate, ice-cream, cookies and sweet pastry

^c^Examples given: soft drinks, juices, and cocoa with added sugar

### Outcomes: children’s food consumption

Children’s food consumption was assessed with a 51-item semi-quantified food frequency questionnaire (FFQ) developed in the DAGIS study and designed to measure particularly the consumption of fruit and vegetables and sugary foods and drinks. Six food groups were assessed: fruit and vegetables; dairy products; fish, meat and eggs; cereal products; beverages; and other foods. An earlier, 47-item version of the same FFQ, excluding the questions on food amounts, showed acceptable test-retest reproducibility [41] and validity for ranking food group consumption against 3-day food records [42]. After the validation and reproducibility studies, 4 food items concerning salty bakery products (not used in this study) and the amount questions (used in this study) were added to the FFQ.

Parents filled the FFQ online. They first reported how often the child had eaten the specific foods or drinks and then answered the questions on amount consumed per day. The FFQ questions on amounts are found in Supplement 1. The questions concerned the previous 7 days. The frequency of consumption was asked about as follows: how often during the seven previous days has the child eaten the listed foods and drinks. Answer options were “not once”, “X times per week” or “X times per day”. The parent either ticked the “not once” option or reported how many times per week or per day the child had consumed the listed foods and beverages. The questions concerning the amounts of foods consumed included between 6 and 12 answer options per food/drink (Supplement 1). Answer options were based on the usual consumption amounts of these foods/drinks found in earlier studies in this age group [43, 44]. Pictures of different portion sizes and their weight were available for each food [45]. Calculating the amounts consumed per day were made as follows: first, eating occasions per week were calculated from the eating frequency questions, with seven consumption frequencies per week used as the maximum. Secondly, the midpoint (except for the second lowest and highest answer options) of each answer option on the amount consumed per day was multiplied by the consumption frequency per week. The amounts used in the formation of the variables are shown in Supplement Table 1. Lastly, the amount consumed per week was divided by seven to calculate the consumption amount per day (g/day).

The FFQ measured only those foods and drinks consumed outside ECEC hours: foods eaten at ECEC centers were left out. In Finland, all municipal ECEC centers provide the foods eaten during childcare hours, and parents cannot reliably report the consumption of these foods.

Four sum variables were formed for this study to represent children’s consumption of FV, sugary everyday foods, sugary treats, and SSB (in g/day). 1) FV consumption comprised the sum of the amount in grams (g) eaten per day of four food items: a) fresh vegetables; b) cooked and canned vegetables; c) fresh fruit; and d) fresh and frozen berries. 2) Consumption of sugary everyday foods comprised the sum of the amount in grams (g) eaten per day of five food items: a) flavored yogurt; b) puddings; c) berry and fruit soups; d) sugared cereals and muesli; and e) berry, fruit and chocolate porridge. 3) Consumption of sugary treats comprised the sum of the amount consumed of five food items: a) ice-cream; b) sweet cookies and cereal bars; c) cakes, cupcakes, and other sweet pastry; d) chocolate; and e) sweets per day. 4) Consumption of SSB comprised the sum consumed per day of three food items: a) flavored and sugar-sweetened milk and plant-based drinks; b) sugar-sweetened juice; c) and sugar-sweetened soft drinks. In the analyses, the difference in follow-up and baseline value of each food variable was used as the outcome variable to represent the change in food consumption.

### Covariates

The child’s age and gender, reported by a parent, were used as covariates in the analyses. The educational level of both parents was sought and categorized as follows: low (including comprehensive, vocational, or high school), medium (including bachelor’s degree or college), or high (including master’s degree or licentiate/doctor). Parental education level (PEL), meaning the highest educational level between the parents, was used in the analyses.

### Statistical analyses

Descriptive statistics of the baseline characteristics of the children according to DOI groups were conducted using IBM SPSS Statistics software version 26.0. The differences in mean between the groups were tested using one-way analysis of variance, while differences in proportions were tested using a chi-squared test. The number of cases in the analyses varied since there was missingness and casewise deletion was used. In mediation analyses, the family was used as a random effect, as there where 19 sibling pairs in the data and we examined the parental DOI.

**Fig. 2:**
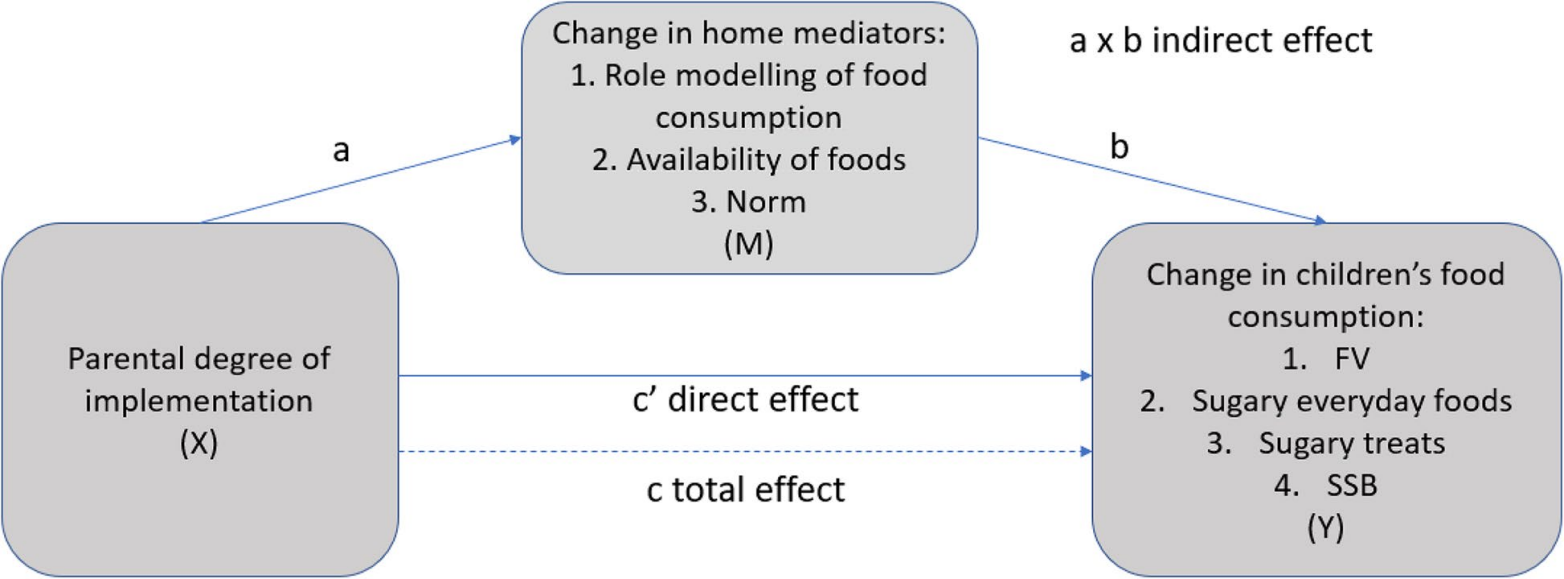
The mediation model for associations between degree of implementation (DOI) of the intervention and change in children’s food consumption Legend: X is the independent variable, Y is the dependent variable and M is the mediator. Path a represents the association between X and M, and path b represents the association between M and Y. c’ path is the direct effect of X on Y controlling for the effect of M and a x b path is the indirect effect of X and Y via M. C path is the total effect of X and M on Y. FV: Fruit and vegetables; SSB: Sugar-sweetened beverages

The mediation analyses were conducted as follows (Fig. 2): First, the effect of the DOI (X) on the change in mediator (M) was estimated (a path). Secondly, the effect of the change in mediator on the change in consumption of FV, sugary everyday foods, sugary treats or SSB (Y) were estimated (b path). Thirdly, the indirect effect of the DOI on the change in food consumption via the proposed mediator (a x b path) was computed. Finally, the direct effect of DOI on change in food consumption, adjusted with the mediator (c’ path) and total effect (c path), were estimated. All mediation analyses were conducted with one mediator at a time, and they were controlled for the baseline values of the mediator and the dependent variable and age, gender and parental education level. Mediation analyses were conducted with statistical software R [46], package “mediation” [47]. The significance level was set at *p* < 0.05.

## Results

**Fig. 3:**
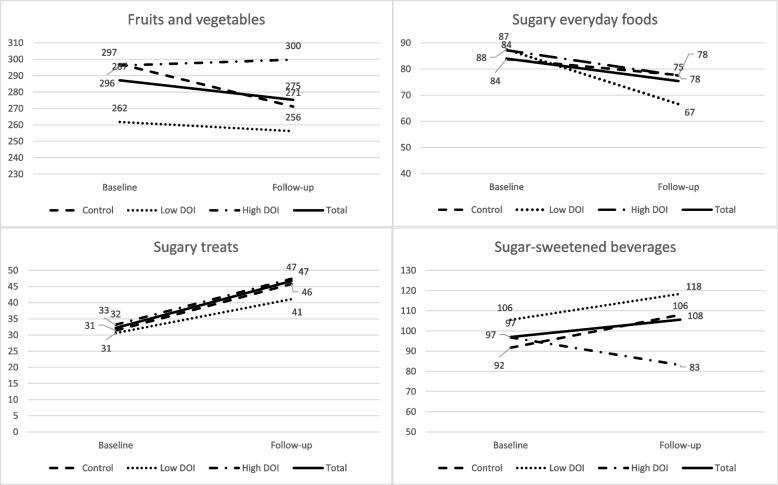
Consumption (g/day) of fruits and vegetables, sugary everyday foods, sugary treats and sugar-sweetened beverages at baseline and at follow-up of the DAGIS intervention

In total, 439 (55% of the consented participants) children had food consumption data and data on home environment from both baseline and follow-up and these children formed the analytic sample. When comparing these children to the whole sample (*n* = 802) according to sociodemographic factors, they differed according to PEL: children in the analytic sample had more highly educated parents than children in the whole sample. No differences according to age or gender were found. The mean age of the children was 5.2 years and 44% of them were girls (Table 3). Over half of the children were in the control group, 20% in low and 22% in high DOI groups. There were no differences in the groups according to child gender, age, living in a two-parent v. one-parent household or parental education level. Children in the control group had more siblings than children in the high DOI group, but no differences between the low and high DOI groups were found. Consumption amounts of FV, sugary everyday foods, sugary treats, and SSB (g/day) at baseline and at follow-up, according to DOI, are shown in Fig. 3. Consumption of FV and SSB was 285 g and 95 g a day respectively at baseline. At baseline, no differences between the groups were observed in the consumption of the studied food groups (not shown in tables). Supplemental Table 2 shows the descriptives of the original mediator variables at baseline and at follow-up. Parents role modelled FV consumption 12.54 times a week in total, whereas most parents reported role modelling of sugary everyday foods, sugary treats and SSB each 1–2 times a week or not at all. The norm for the number of portions of FV per day was ca 4, whereas for sugary foods and beverages it was less than one for each. The descriptives of mediators and food consumption variables used in the analyses (namely the difference in follow-up and baseline values) are shown in Table 4.

**Table 3:** Descriptives of the study sample at baseline, according to degree of implementation (DOI)

n	Total	control	low DOI	high DOI	p*
	439	253	82	98	
**Age, years**^**c**^	5.2 (1.0)	5.2 (1.1)	5.2 (1.0)	5.1 (0.9)	0.56^a^
**Gender, girls**^**d**^	194 (44.2%)	96 (40.9%)	39 (47.6%)	50 (51.0%)	0.20^b^
**Living in two-parent household**^**d**^	393 (90.3%)	214 (92.2%)	73 (89.0%)	85 (87.6%)	0.38
**Number of siblings**^**c**^	1.2 (0.9)	1.3 (0.9)^e^	1.2 (0.8)	1.0 (0.8)^e^	0.04
**PEL**^**d**^					0.30^b^
Low	116 (26.5)	53 (22.6)	21 (25.9)	31 (31.6)	
Medium	210 (47.9)	113 (48.1)	39 (48.1)	48 (49)	
High	112 (25.6)	69 (29.4)	21 (25.9)	19 (19.4)	

*DOI* degree of implementation, *PEL* parental education level (highest in the family); * for differences between control group, low and high DOI groups

^a^analysis of variance

^b^chi-square test

^c^mean (SD)

^d^n (%)

^e^Bonferroni post-hoc test p 0.04

**Table 4:** The change (difference between follow-up and baseline values) in children’s food consumption variables and mediators, in total and according to DOI groups

	n	Mean (SD)			
		Total	Control	Low DOI	High DOI
		432–439	233–235	82	98
**Change in consumption of (g/day)**	**FV**	-6.74 (141.46)	-23.47 (134.44)	5.38 (157.04.)	3.63 (136.03)
	**Sugary everyday foods**	-11.00 (75.65)	-7.77 (74.86)	-23.60 (77.13)	-9.75 (76.63)
	**Sugary treats**	14.75 (39.21)	14.47 (37.22)	10.90 (35.76)	14.17 (38.95)
	**SSB**	11.41 (111.80)	18.47 (114.31)	15.40 (111.36)	-13.74 (98.62)
**Change in role modelling**	**FV**	-0.14 (5.36)	-0.28 (5.36)	0.46 (5.56)	-0.34 (5.41)
	**Sugary everyday foods**	-0.04 (1.78)	-0.11 (1.85)	0.12 (1.89)	-0.01 (1.56)
	**Sugary treats**	0.14 (1.55)	0.07 (1.75)	0.20 (1.30)	0.20 (1.28)
	**SSB**	0.09 (1.42)	0.07 (1.48)	0.09 (1.63)	0.03 (1.09)
**Change in availability**	**FV**	0.05 (0.55)	0.06 (0.57)	0.01 (0.49)	0.08 (0.58)
	**Sugary everyday foods**	0.02 (0.54)	0.09 (0.54)	-0.15 (0.51)	-0.06 (0.55)
	**Sugary treats**	0.01 (0.63)	0.01 (0.64)	-0.05 (0.64)	0.01 (0.60)
	**SSB**	0.09 (0.81)	0.12 (0.86)	0.05 (0.68)	-0.03 (0.81)
Change in norm*	**FV**	0.00 (1.81)	-0.15 (1.71)	0.04 (2.08)	0.18 (1.81)
	**Sugary everyday foods**	0.06 (0.63)	0.00 (0.63)	-0.10 (0.63)	-0.17 (0.58)
	**Sugary treats**	-0.01 (0.26)	-0.01 (0.23)	-0.04 (0.30)	0.00 (0.29)
	**SSB**	-0.00 (0.45)	0.03 (0.44)	-0.04 (0.53)	-0.07 (0.39)

*DOI* degree of implementation, *FV* Fruit and vegetables, *SSB* Sugar-sweetened beverages

*Parental opinion on the suitable number of portions per day for children aged 3–6 years

The results of the mediation analyses are shown in Table 5. A direct effect of DOI was found for SSB: children in the high DOI group reduced their consumption of SSB compared to the control group (results when adjusted with parental role modelling: B -27.71, 95% CI -49.05, -4.80, p 0.02). Total effect was B -28.71 (95% CI -49.05, -5.61) (not shown in Table). No other direct effects were found. In the high DOI group there was an indirect effect via a change in norm (parental opinion on the suitable number of portions) leading to increased FV consumption (a x b B 4.31, 95% CI 0.23,  10.59, p 0.03). The total effect was not found (B 20.52, 95% CI -8.51,  49.77, p 0.17). In addition, in the low DOI group, there was an indirect effect via decreased availability, leading to decreased sugary everyday food consumption (a x b B -2.17, 95% CI -5.09, -0.09, p 0.04). The total effect was also found (B -15.06, 95% CI -30.96,  -0.58, p 0.04) (not shown in Table).

**Table 5:** Mediation analysis results of the degree of implementation of the DAGIS intervention on children’s consumption of FV, sugary everyday foods, sugary treats, and SSB. Single mediator models, control group as the reference

	a path^**1**^				b path^**2**^		a x b path^**3**^				Direct effect^**4**^			
	Low DOI		High DOI				Low DOI		High DOI		Low DOI	High DOI		
	B (SE)	p	B (SE)	p	B (SE)	p	B (95% CI)	p	B (95% CI)	p	B (95% CI)	p	B (95% CI)	p
**FV,** ***n*** **= 406–412**														
Availability	-0.09 (0.06)	0.14	0.07 (0.06)	0.21	**44.90 (12.65)**	**> 0.001**	-4.24 (-11.38, 1.45)	0.14	3.10 (-2.03,9.26)	0.28	13.43 (-16.75,45.03)	0.39	17.87 (-11.26, 45.59)	0.22
Parental role modelling	-0.27 (0.59)	0.64	0.52 (0.55)	0.38	**7.08 (1.36)**	**> 0.001**	-1.96 (-11.00, 5.85)	0.65	3.72 (-3.95, 12.19)	0.34	4.60 (-28.06, 38.90)	0.79	18.29 (-12.44, 47.68)	0.22
Norm	-0.02 (0.20)	0.92	**0.43 (0.19)**	**0.02**	**9.93 (4.09)**	**0.02**	-0.31 (-4.91, 4.29)	0.85	**4.31 (0.23, 10.59)**	**0.03**	10.90 (-20.98, 41.89)	0.5	16.22 (-13.06, 45.01)	0.27
**Sugary everyday foods,** ***n*** **= 409–413**														
Availability	**-0.13 (0.06)**	**0.03**	-0.11 (0.06)	0.06	**18.44 (6.39)**	**> 0.01**	**-2.17 (-5.09, -0.09)**	**0.04**	-1.93 (-4.71, 0.09)	0.07	-12.90 (-28.34, 2.11)	0.09	6.23 (-7.63, 21.03)	0.38
Parental role modelling	0.13 (0.19)	0.49	0.06 (0.18)	0.73	**5.61 (2.05)**	**> 0.01**	0.73 (-1.46, 3.37)	0.52	0.35 (-1.82, 2.60)	0.74	-11.87 (-27.21, 2.44)	0.12	3.24 (-10.77, 17.46)	0.66
Norm	-0.02 (0.07)	0.75	**-0.13 (0.06)**	**0.04**	4.69 (6.00)	0.43	-0.07 (-1.14, 0.88)	0.92	-0.55 (-2.34, 0.94)	0.48	-10.80 (-25.90, 5.04)	0.18	3.03 (-10.82, 17.48)	0.69
**Sugary treats,** ***n*** **= 412–414**														
Availability	-0.02 (0.06)	0.78	0.01 (0.06)	0.86	**9.41 (3.31)**	**> 0.01**	-0.15 (-1.41, 1.05)	0.79	0.10 (-1.17, 1.39)	0.85	-3.50 (-12.04, 5.45)	0.41	2.42 (-5.73, 10.49)	0.56
Parental role modelling	0.09 (0.16)	0.57	0.12 (0.15)	0.44	2.35 (1.34)	0.08	0.22 (-0.64, 1.29)	0.57	0.26 (-0.43, 1.37)	0.5	-3.03 (-10.91, 5.85)	0.47	1.75 (-6.27, 9.63)	0.69
Norm	-0.00 (0.02)	0.88	-0.02 (0.02)	0.36	7.48 (8.25)	0.37	-0.03 (-0.69, 0.58)	0.94	-0.15 (-0.89, 0.34)	0.6	-3.84 (-12.33, 4.44)	0.37	3.95 (-4.36, 12.28)	0.35
**SSB, n = 409–413**														
Availability	-0.00 (0.09)	0.98	-0.16 (0.08)	0.06	**18.30 (6.66)**	**> 0.01**	0.07 (-3.76, 3.56)	0.99	-2.88 (-7.23, 0.14)	0.06	17.80 (-7.02, 42.16)	0.15	**-26.49 (-48.61, -5.05)**	**0.02**
Parental role modelling	0.01 (0.15)	0.94	-0.09 (0.13)	0.53	**11.85 (4.19)**	**> 0.01**	0.05 (-3.90, 3.81)	0.99	-1.03 (-4.86, 2.45)	0.53	19.93 (-2.90, 42.46)	0.1	**-27.71 (-49.05, -4.80)**	**0.02**
Norm	0.06 (0.04)	0.17	-0.05 (0.04)	0.23	**33.67 (14.01)**	**0.02**	2.24 (-1.07, 6.90)	0.18	-1.63 (-5.56, 1.08)	0.26	11.77 (-13.73, 36.12)	0.35	**-27.10 (-49.20, -4.90)**	**0.02**

Significant associations bolded; DOI: degree of implementation. *SE* standard error, *FV* Fruit and vegetables, *SSB* Sugar-sweetened beverages

^a^path adjusted for: age, gender, parental education level, and baseline value of the mediator

^b^path adjusted for: age, gender, parental education level, and baseline values of the mediator and outcome

^1^effect of the independent variable on the mediator (difference of follow-up and baseline values of the mediator)

^2^effect of the mediator on the outcome (difference of follow-up and baseline values of the outcome)

^3^effect of the independent variable on the outcome via the mediator

^4^effect of the independent variable on the outcome when adjusting for the mediator

## Discussion

This study examined whether the parental DOI of a family-involved preschool intervention impacted children’s consumption of FV, sugary everyday foods, sugary treats or SSB via home mediators: food availability, parental role modelling and norm. We found that consumption of SSB decreased in the high DOI group compared to the control group, but the decrease was not mediated by the studied mediators. Also, in the low DOI group, consumption of sugary everyday foods decreased via decreased availability compared to the control group. Yet, in the high DOI group, a change in norm (increase in parental view on the suitable number of portions of FV for children) mediated a positive effect on children’s FV consumption compared to the control group.

Similarly to our results, previous interventions on children’s SSB consumption have mainly succeeded in decreasing children’s SSB consumption [17, 18]. Still, it is notable that the found effect was not mediated by food availability, parental role modelling or norm, although the indirect effect via decreased availability was borderline statistically significant (p 0.06). A change in food availability is a potent mediator in dietary interventions found in other studies too [23]. Further mediation analyses with other possible mediators, such as rules and restrictions, are warranted. The effectiveness of the DAGIS intervention on children’s EBRBs and self-regulation skills has been examined already by Ray et al. [32] finding no intervention effect on the intake frequency of sugary everyday foods and drinks combined together. This study builds on these results by further studying the role of DOI and possible mediated effects. The difference in the results between the studies emphasizes the importance of examining DOI together with intervention effectiveness as we found an effect in the high DOI group. In addition, in this study we examined slightly different food groups, as well as different food consumption outcomes: frequency v. amount.

Concerning sugary everyday foods, we found an indirect and total effect of low DOI on reduced consumption of sugary everyday foods via decreased availability. In addition, in the high DOI group, the same indirect effect was borderline significant (p 0.07). These results correspond to previous results on intervention effects on children’s food consumption via home availability, although the studied food group in this study differs somewhat from previous research [23, 48]. All in all, in this age group, intervention effects may be easier to obtain in reducing the consumption of unhealthy foods or beverages than increasing consumption of healthy foods or beverages, as restricting home food availability or other restrictions can have immediate effects on consumption.

We found an indirect effect of high DOI on children’s FV consumption via parental norm, meaning that in the high DOI group the parental view on the suitable number of daily portions of FV for children increased and this was associated with increased FV consumption among children. No total effect on children’s FV consumption was found, but the existence of indirect effects in the absence of total effect is not exceptional in mediation analyses [49]. Previous studies have mainly found the availability and parental offering of FV to mediate intervention effects on children’s FV consumption [21]. In this study, the lack of effects on availability of FV at home could be due to the already high availability at baseline. Previous studies on the mediating role of norms on children’s food consumption were not found. Instead, Lambrinou et al. [48] found there was a mediating effect of parental knowledge, attitude and rules on children’s consumption of snacks (including FV and sweet and savoury snacks), and some mediating effects were also found on parental self-efficacy too [21]. The lack of total effects on children’s FV consumption may be due to the relatively short timeframe: the duration of the FV theme period of the intervention was 5 weeks and this might be insufficient time to increase the liking of fruits and vegetables that might be necessary in order to increase their consumption [50]. Repeated exposure to vegetables in order to increase liking is a method that many interventions use to promote vegetable intake [51].

Most of the mediators in this study had an effect on children’s consumption of FV, sugary everyday foods, sugary treats, and SSB, implying that the intervention targeted the determinants of children’s food consumption. Yet, the intervention, even when implemented to a greater extent, impacted only a few of the mediators, which was similar to Lambrinou et al.’s study [48]. A broader spectrum of possible mediators, such as rules, practices, and self-efficacy, might have revealed effects not found here, as we only used three possible mediators in our analyses. The limited amount of effects on the mediators, as well as on children’s food consumption, might have been due to insufficient time for change to take place, or perhaps having too many objectives in the intervention, which could have left the parents unable to concentrate for long enough on one health behavior to be able to bring about change.

In a review of preschool intervention effects on children’s food consumption it was found that parental involvement in intervention development or activities was a unifying factor in many successful interventions [17]. In the DAGIS intervention, much effort had been put into involving and activating parents in the intervention, for example by reading stories about health behaviors to the child and then discussing them, family activating games about health behaviours and family activities at the ECEC center. Still, the impact on family mediators was also quite modest in the high DOI group; a lack of motivation among parents to instigate change may be one explanation. Between 60 and 82% of parents reported being pleased with their child’s consumption of vegetables, fruit, sugary everyday foods, sugary treats and SSB at baseline, which might indicate a lack of motivation to promote change. All in all, as improving DOI is essential for gaining more intervention effects, particular effort should be put into involving and motivating parents in interventions that require parental actions. Methods to improve parental DOI may include increasing parental motivation, improving the easiness of implementation, and support [52–54].

In the long term, the DAGIS intervention aims at reducing the risk of overweight among children, but a follow-up of potentially several years would be needed to elicit such effect. Results of this study set hopes for this possible effect particularly in the high DOI group, as interventions reducing SSB consumption have succeeded in reducing weight among adolescents [55]. Concerning FV, to the best of our knowledge, no studies on long term health effects of childhood FV consumption interventions is found, but increasing FV consumption has been found to be effective in primary prevention of cardiovascular diseases among adults [56] Additionally, in observational studies it has been found that high consumption of FV has been found to reduce the risk of several chronic diseases [4, 6, 57]. Despite several partly overlapping theoretical frameworks in the field of process evaluation [26, 38, 58, 59], the concrete assessment of process evaluation depends on the intervention and can be done in many different ways. In addition, the summarizing and rating of DOI in one score is somewhat arbitrary, despite trying to consider all or some aspects of a theoretical framework. In the DOI variable, we gave weight mainly to parents’ active implementation of the intervention activities, as we stressed dose received – exposure dimension in the scoring. We decided to do this as we considered this dimension to be the most important factor in achieving intervention effects. It can be speculated whether different results would have been obtained with a different DOI variable and/or scoring, e.g. by giving more weight to parents’ satisfaction or fidelity. The used DOI variable included both more objective measures, such as those in the dose delivered dimension, and more subjective measures, such as those included in the satisfaction dimension. The importance of these dimensions for the effectiveness of the intervention may vary and can be a subject of further studies. The cutoff point of 10.1 points (median) of a maximum of 18.5 points was used in order to divide the intervention group into low and high DOI groups, which meant that a little over half of the intervention activities, satisfaction and fidelity points needed to be achieved in order to be included in the high DOI group. According to Durlak and DuPre [26], in the real world it is unrealistic to assume total compliance and 60% implementation is enough to achieve effects, but this, of course, depends on the intervention. The process evaluation data in this study were entirely self-reported by parents, which might be prone to bias, as researcher-reported process evaluation data are associated with intervention effects more often than self-reported data [24]. Qualitative process evaluation data, more data-driven statistical methods, and examining the dimensions of the DOI variable separately would give valuable information on different aspects of DOI and factors affecting it.

The use of a semi-quantified FFQ in the study requires further discussion. The questions on amounts of foods in the FFQ have not been validated, and thus caution is required when interpreting this data. When comparing the consumption amounts in this study to similar food consumption variables, which are based on data from 3-day food records of Finnish children in the same age group, it seems that the FFQ overestimates the consumption of FV (287 vs. 177 g), but amounts of SSB, sugary everyday foods and sugary treats seem similar [60]. Instead, the test-retest reproducibility of the FFQ has been studied, with most questions showing moderate to good test-retest reproducibility [41]. In addition, the reproducibility was even slightly better for the questions on amounts than for the questions about frequency of consumption. In future, the use of a validated semi-quantified FFQ may elicit a larger variation in the data and bring forward changes in consumption not detectable with ordinary FFQs, which favors their use specifically in intervention studies.

### Strengths and weaknesses

The strengths of this study include extensive use of data when combining process evaluation data with intervention effectiveness data and possible mediators. Another strength was that the mediation model was based on the Intervention Mapping framework [25] and on the logic model of change of the DAGIS intervention [31], and that DOI was measured with a vast and comprehensive data according to Saunders’ framework [38]. The participation rate of the study was fairly good (47%), but as the sample consisted of children cared for in ECEC centers in two municipalities in Southern Finland, it is not representative of all Finnish 3–6-year-olds. Still, according to the parents’ educational level, and the fact that all public ECEC centers in the other municipality participated in the study, the sample appears rather diverse. A weakness in this study is that the number of the targeted health behaviors and the timetable of the intervention might have impacted the found effects: the intervention included many aims and targeted health behaviors, which may have been too burdensome for the participants, hampering possible behavior changes. Also, the FV period of the intervention occurred during the winter, while the sugar period took place immediately before the follow-up measurements in spring. This might explain why more effects were found for sugary foods and beverages. As discussed above, another weakness was that the validity of the questions on consumption amounts in the FFQ is unknown. Also, the use of a dichotomized DOI variable may be considered as a weakness as categorization may cause losing power. However, we used a categorized DOI variable as we wanted to use the control group as the reference.

## Conclusions

We found a decreasing intervention effect on children’s SSB consumption among families where the parents implemented the intervention to a large extent. This impact was not mediated by the studied mediators. In addition, low DOI was associated with reduced consumption of sugary everyday foods via decreased availability at home and there was an indirect effect in the high DOI group leading to increased FV consumption via increased parental view on the suitable amount of FV for children. In conclusion, this study adds knowledge about the importance of DOI for the intervention effectiveness and encourages to put effort to improving DOI in health promotion interventions implemented by parents. In addition, considering possible mediators of the intervention was important as this provided further insights into the intervention effects and mediation paths. In future, a closer look at distinct intervention activities could elicit more specific knowledge about which components of the intervention were effective. In addition, the determinants of DOI should be studied in order to gain an understanding of which factors could increase parental DOI. This could help future intervention developers in developing effective interventions.

## Supplementary Information

**Additional file 1.**

## Data Availability

The datasets used and/or analysed during the current study are available from the corresponding author on reasonable request.

## Abbreviations

EBRB: Energy balance-related behavior
ECEC: Early childhood education and care
DOI: Degree of implementation
FFQ: Food frequency questionnaire
FV: Fruit and vegetables
g: Gram
PEL: Parental education level
SSB: Sugar-sweetened beverages
