# Supplementary material for: Effects of the DAGIS randomized controlled trial on home environment and children’s food consumption according to the degree of implementation
Source: BMC Public Health. 2022 Dec 5;22:2268. doi: 10.1186/s12889-022-14639-y (PMC9720976; doi:10.1186/s12889-022-14639-y)
Supplement: Supplementary file 1 — Additional file 1. [file 12889_2022_14639_MOESM1_ESM.docx]

Supplemental Table 1. The amount questions and their answer options on consumption of fruits, vegetables, sugary everyday foods, sugary treats and sugar-sweetened beverages in the FFQ used in the DAGIS intervention study.

| **Question** | **Examples given** | **Answer options** | **Amount used to calculate consumption amounts** |
| --- | --- | --- | --- |
| **Fruits and vegetables** |  |  |  |
| 1. Think about the previous week and the days your child ate **fresh vegetables**. On average, how much did your child eat fresh vegetables (e.g. lettuce, grated carrots, tomatoes, cucumbers) per day? The question concerns foods eaten outside of preschool. | Cucumber slice = 5 g  Cherry tomato = 15 g  1 dl grated carrot = 40 g  Medium tomato = 125 g | 1. None 2. Less than 30 g 3. 30-59 g 4. 60-89 g 5. 90-119 g 6. 120-149 g 7. 150-179 g 8. 180-209 g 9. 210-239 g 10. 240-269 g 11. 270 g or more | 1. 0 2. 20 g 3. 45 g 4. 75 g 5. 105 g 6. 135 g 7. 165 g 8. 195 g 9. 225 g 10. 255 g 11. 284 g |
| 2. Think about the previous week and the days your child has eaten **cooked vegetables and/or canned vegetables**. On average, how much did your child eat cooked vegetables and/or canned vegetables (as a side dish, as part of dishes, e.g. mushrooms) per day? The question concerns foods eaten outside of preschool. | Mushroom slice = 6 g  1 dl frozen vegetables = 60 g | Same as in question 1 | Same as in question 1 |
| 3. Think about the previous week and the days your child ate **fresh fruit**. On average, how much did your child eat fresh fruit per day? The question concerns foods eaten outside of preschool. | Medium-sized banana peeled = 120 g  Medium orange peeled = 190 g  Medium apple with peel = 200 g | Same as in question 1 | Same as in question 1 |
| 4. Think about the previous week and the days your child ate **berries**. On average, how much did your child eat berries (fresh or frozen) per day? The question concerns foods eaten outside of preschool. | Medium strawberry (fresh) = 8 g  1 dl fresh strawberries = 50 g  1 dl blueberries = 60 g | 1. None 2. Less than 35 g 3. 35-69 g 4. 70-104 g 5. 105-139 g 6. 140-174 g 7. 175 g or more | 1. 0 g 2. 23 g 3. 52 g 4. 87 g 5. 122 6. 157 g 7. 184 g |
| **Sugary everyday foods** |  |  |  |
| 5. Think about the previous week and the days your child ate **berry and fruit fools and thickened soups** with added sugar. On average, how much did your child eat berry and fruit fools and thickened soups with added sugar per day? The question concerns foods eaten outside of preschool. | 1 dl fool = 100 g | 1. None 2. Less than 50 g 3. 50-99 g 4. 100-149 g 5. 150-199 g 6. 200 g or more | 1. 0 g 2. 33 g 3. 75 g 4. 125 g 5. 175 g 6. 210 g |
| 6. Think about the previous week and the days your child ate **flavored yogurt and/or quark.** On average, how much did your child eat flavored yogurt or quark (also plant-based, e.g. Champion Yogurt, Yosa) per day? The question concerns foods eaten outside of preschool. | 1 dl yogurt = 100 g  Yogurt jar = 200 g | 1. None 2. Less than 65 g 3. 65-129 g 4. 130-194 g 5. 195 g or more | 1. 0 g 2. 43 g 3. 97 g 4. 162 g 5. 205 g |
| 7. Think about the previous week and the days your child ate **puddings**. On average, how much did your child eat puddings (*) per day? The question concerns foods eaten outside of preschool. | 1. dl pudding = 100 g   * | Same as in question 6 | Same as in question 6 |
| 8. Think about the previous week and the days your child ate **sugary cereals and/or muesli**. On average, how much did your child eat sugary cereals and/or muesli (e.g. honey or cocoa cereals, muesli) per day? The question concerns foods eaten outside of preschool. | 1 tablespoon muesli = 7.5 g  1 dl breakfast cereals (e.g. Corn Flakes) = 13 g | 1. None 2. Less than 15 g 3. 15-29 g 4. 30-44 g 5. 45 g or more | 1. 0 g 2. 10 g 3. 22 g 4. 37 g 5. 47 g |
| 9. Think about the previous week and the days your child ate **berry, fruit and/or chocolate porridge**. On average, how much berry, fruit and/or chocolate porridge (containing added sugar) did your child eat per day? The question concerns foods eaten outside of preschool. | 1 dl porridge = 100 g  1 sachet of instant porridge prepared according to instructions = 170 g | 1. None 2. Less than 50 g 3. 50-99 g 4. 100-149 g 5. 150-199 g 6. 200-249 g 7. 250-299 g 8. 300 g or more | 1. 0 g 2. 33 g 3. 75 g 4. 125 g 5. 175 g 6. 225 g 7. 275 g 8. 315 g |
| **Sugar-sweetened beverages** |  |  |  |
| 10. Think about the previous week and the days your child consumed **flavored and** **sweetened milk and/or plant-based drinks**. On average, how much did your child consume flavored and sweetened milk and/or plant-based beverages (e.g. cocoa, soya drink) per day? The question concerns foods eaten outside of preschool. |  | 1. None 2. Less than 1 dl 3. 1-1.9 dl 4. 2-2.9 dl 5. 3-3.9 dl 6. 4-4.9 dl 7. 5-5.9 dl 8. 6-6.9 dl 9. 7-7.9 dl 10. 8-8.9 dl 11. 9-9.9 dl 12. 10 dl or more | 1. 0 g 2. 67 g 3. 145 g 4. 245 g 5. 345 g 6. 445 g 7. 545 g 8. 645 g 9. 745 g 10. 845 g 11. 945 g 12. 1050 g |
| 11.Think about the previous week and the days your child has been drinking **sugary juice**. On average, how much did your child drink sugary juice per day? The question concerns foods eaten outside of preschool. | Juice box = 2 dl | Same as in question 10 | Same as in question 10 |
| 12. Think about the previous week and the days your child drank **sugary soft drinks**. On average, how much did your child drink sugary soft drinks (e.g. Coca-Cola, Jaffa) per day? The question concerns foods eaten outside of preschool. | Glass bottle or small can = 2.5 dl  Normal can = 3.3 dl  Small plastic bottle = 5 dl | Same as in question 10 | Same as in question 10 |
| **Sugary treats** |  |  |  |
| 13. Think about the previous week and the days your child ate **ice cream**. ON average, how much did your child eat ice cream (*) a day? The question concerns foods eaten outside of preschool. | 1 dl ice cream = 50 g  Medium size ice cream stick = 60 g  Medium size ice cream cone = 115 g | 1. None 2. Less than 50 g 3. 50-99 g 4. 100-149 g 5. 150-199 g 6. 200 g or more | 1. 0 g 2. 33 g 3. 75 g 4. 125 g 5. 175 g 6. 210 g |
| 14. Think about the previous week and the days your child ate **sweet biscuits and/or snack biscuits**. On average, how much did your child eat sweet biscuits and/or snack biscuits per day? The question concerns foods eaten outside of preschool. | * | 1. None 2. Less than 5 g 3. 5-14 g 4. 15-29 g 5. 30-44 g 6. 45-59 g 7. 60 g or more | 1. 0 g 2. 3 g 3. 9 g 4. 22 g 5. 37 g 6. 52 g 7. 63 g |
| 15. Think about the previous week and the days your child ate **cake, muffins, buns, pies and/or other sweet pastries**. On average, how much did your child eat cake, muffins, buns, pie and/or other sweet pastries per day? The question concerns foods eaten outside of preschool. | Medium wrapped cake slice = 20 g  Medium muffin = 50 g  Medium stuffed cake piece = 70 g | 1. None 2. Less than 35 g 3. 35-69 g 4. 70-104 g 5. 105-139 g 6. 140-174 7. 175 g or more | 1. 0 g 2. 23 g 3. 52 g 4. 87 g 5. 122 g 6. 157 g 7. 184 g |
| 16. Think about the previous week and the days your child ate **chocolate**. How much, on average, did your child eat chocolate (e.g. chocolate raisins, chocolates, milk chocolate) per day?  The question concerns foods eaten outside of preschool. | Chocolate candy = 8 g  * | 1. None 2. Less than 15 g 3. 15-24 g 4. 25-49 g 5. 50-74 g 6. 75-99 g 7. 100-124 g 8. 125 g or more | 1. 0 g 2. 10 g 3. 20 g 4. 37 g 5. 62 g 6. 87 g 7. 112 g 8. 131 g |
| 17. Think about the previous week and the days your child ate **sweets**. How much, on average, did your child eat sweets (e.g. salty liquorice or fruit candies, loose candies, lollipops) per day? The question concerns foods eaten outside of preschool. | A piece of toffee = 5 g  Licorice bar (filled) = 11 g  Lollipop = 12 g | Same as in question 16. | Same as in question 16. |

*Examples of common Finnish brand names and their weights are given.

Supplemental Table 2. Descriptives of the mediators at baseline and at follow-up in total sample and according to degree of implementation (DOI).

|  | | **Baseline** | | | | | | **Follow-up** | | | |
| --- | --- | --- | --- | --- | --- | --- | --- | --- | --- | --- | --- |
|  | | **Total** | **Control** | **Low DOI** | **High DOI** | | **P^a^** | **Total** | **Control** | **Low DOI** | **High DOI** |
| n | | 438-439 | 235 | 82 | 97-98 | |  | 438-429 | 235 | 82 | 97–98 |
|  | | **Percentages %** | | | | | | **Percentages %** | | | |
| **Role modelling consumption of sugary everyday foods** | | | | | | | | | | | |
| not at all | | 33.9 | 34.0 | 34.1 | 33.7 | | 0.89 | 35.0 | 34.9 | 35.4 | 37.1 |
| 1–2 times/week | | 46.0 | 44.3 | 48.8 | 49.0 | |  | 45.1 | 46.4 | 45.1 | 42.3 |
| 3–4 times/week | | 13.2 | 14.9 | 11.0 | 10.2 | |  | 13.0 | 11.5 | 12.2 | 14.4 |
| 5–6 times/week | | 3.2 | 3.0 | 3.7 | 3.1 | |  | 3.0 | 3.8 | 3.7 | 1 |
| once everyday | | 3.0 | 2.6 | 2.4 | 4.1 | |  | 3.7 | 3.4 | 2.4 | 5.2 |
| more than once a day | | 0.7 | 1.3 | 0 | 0 | |  | 0.2 | 0 | 1.2 | 0 |
| **Role modelling consumption of sugary treats** | | | | | | | | | | | |
| not at all | | 25.5 | 26.4 | 23.2 | 22.4 | | 0.23 | 23.3 | 25.1 | 19.5 | 22.7 |
| 1–2 times/week | | 59.2 | 56.3 | 64.6 | 65.3 | |  | 57.1 | 55.3 | 61.0 | 58.8 |
| 3–4 times/week | | 11.8 | 13.6 | 7.3 | 10.2 | |  | 15.1 | 14.5 | 15.9 | 13.4 |
| 5–6 times/week | | 2.1 | 3.0 | 2.4 | 1.0 | |  | 1.8 | 3.4 | 0 | 0 |
| once everyday | | 0.7 | 0 | 2.4 | 1.0 | |  | 2.7 | 1.7 | 3.7 | 5.2 |
| more than once a day | | 0.7 | 0.9 | 0 | 0 | |  | 0 | 0 | 0 | 0 |
| **Role modelling SSB consumption** | | | | | | | | | | | |
| not at all | | 63.1 | 60.4 | 65.9 | 66.3 | | 0.24 | 59.7 | 57.4 | 63.4 | 63.3 |
| 1–2 times/week | | 29.8 | 30.2 | 29.3 | 28.6 | |  | 33.0 | 34.9 | 30.5 | 30.6 |
| 3–4 times/week | | 6.2 | 8.9 | 2.4 | 4.1 | |  | 5.2 | 5.1 | 2.4 | 6.1 |
| 5–6 times/week | | 0.2 | 0 | 1.2 | 0 | |  | 0.9 | 0.9 | 2.4 | 0 |
| once everyday | | 0.7 | 0.4 | 1.2 | 1.0 | |  | 1.1 | 1.7 | 1.2 | 0 |
| more than once a day | | 0 | 0 | 0 | 0 | |  | 0 | 0 | 0 | 0 |
|  | | **Baseline** | | | | | | **Follow-up** | | | |
|  | | **Mean (SD)** | | | | | | **Mean (SD)** | | | |
|  | | **Total** | **Control** | **Low DOI** | **High DOI** | **p^b^** | | **Total** | **Control** | **Low DOI** | **High DOI** |
| **Role modelling** | **FV** | 12.54 (5.29) | 12.77 (5.48) | 11.33^c^ (5.32) | 13.53^c^ (4.55) | 0.02 | | 12.39 (5.41) | 12.49 (5.35) | 11.77 (5.46) | 13.18 (5.63) |
| **Availability** | **FV** | 4.06 (0.62) | 4.09 (0.62) | 4.00 (0.62) | 4.11 (0.60) | 0.46 | | 4.11 (0.66) | 4.15 (0.65) | 4.01 (0.71) | 4.19 (0.61) |
|  | **Sugary everyday foods** | 2.55 (0.69) | 2.50 (0.65) | 2.63 (0.66) | 2.50 (0.57) | 0.25 | | 2.57 (0.67) | 2.59 (0.63) | 2.59 (0.61) | 2.46 (0.56) |
|  | **Sugary treats** | 2.86 (0.68) | 2.86 (0.73) | 2.93 (0.66) | 2.82 (0.62) | 0.60 | | 2.87 (0.65) | 2.88 (0.67) | 2.88 (0.60) | 2.84 (0.66) |
|  | **SSB** | 2.40 (0.88) | 2.41 (0.91) | 2.44 (0.85) | 2.34 (0.87) | 0.70 | | 2.49 (0.91) | 2.53 (0.93) | 2.50 (0.93) | 2.31 (0.84) |
| **Norm*** | **FV** | 4.15 (1.90) | 4.14 (1.81) | 4.07 (2.00) | 4.41 (2.11) | 0.42 | | 4.13 (1.92) | 3.98 (1.79) | 4.06 (2.01) | 4.54 (2.12) |
|  | **Sugary everyday foods** | 0.94 (0.62) | 0.94 (0.65) | 0.96 (0.61) | 0.95 (0.59) | 0.99 | | 0.90 (0.72) | 0.98 (0.84) | 0.85 (0.57) | 0.77 (0.51) |
|  | **Sugary treats** | 0.37 (0.30) | 0.39 (0.32) | 0.39 (0.25) | 0.33 (0.29) | 0.25 | | 0.36 (0.25) | 0.37 (0.23) | 0.37 (0.29) | 0.33 (0.27) |
|  | **SSB** | 0.49 (0.48) | 0.44^d^ (0.42) | 0.63^d^ (0.62) | 0.48 (0.47) | 0.01 | | 0.49 (0.47) | 0.48 (0.41) | 0.59 (0.57) | 0.43 (0.49) |

^a^ chi-square test for differences between control group, low and high DOI groups;

^b^ analysis of variance for differences between control group, low and high DOI groups;

*parent’s view on suitable number of portions per day for 3-6-year-old children.

^c^ Bonferroni post-hoc test p 0.02

^d^ Bonferroni post-hoc test p 0.01
